# Editorial

**Published:** 2013-09-06

**Authors:** Shibal Bhartiya, Tarek Shaarawy, Tanuj Dada

The more extensive a man’s knowledge of what has been done, the greater will be his power of knowing what to do

—Benjamin Disraeli

The editorial team of the Journal of Current Glaucoma Practice takes great pleasure in bringing to you this issue of the Journal. We hope you will find this issue insightful and relevant to your practice of glaucoma.

In the basic diagnostics section, Gandhi et al emphasize on the technique of examination of the disk parameters, including size, shape, neuroretinal rim shape and pallor, size, configuration and depth of the optic cup and ancillary findings to help the clinician differentiate between glaucomatous and nonglaucomatous optic neuropathy.

The pediatric uveitic glaucomas present a great diagnostic and therapeutic challenge for the glaucomatologist. Kaur et al review the clinical spectrum of this disease subgroup along with its risk factors and treatment outcomes.

Ariga et al elucidate the common and, yet often (overlooked?), pseudoexfoliation syndrome and chronicle the pearls and pitfalls of it’s diagnosis in an illustrated review.

The demographic characteristics of cataract and glaucoma overlap and so the two diseases are often coexistent. Kuldev Singh critically analyzes cataract surgery in glaucoma patients in terms of intraocular pressure control and beyond, presenting evidence that the most important reasons for performing early cataract surgery in those with glaucoma may have little to do with intraocular pressure (IOP) lowering. Apart from the two diseases being coexistent and temporally separated by way of severity, cataract formation is a known complication of glaucoma surgery. Management of cataract in the patients with previous glaucoma filtering surgery is a challenging proposition for any surgeon, as the surgery can lead to several complications in the already compromised eye. Dada et al highlight the intra- and postoperative measures that may increase the chances of bleb survival following cataract surgery in eyes with previous glaucoma filtering surgeries.

Normal tension glaucoma presents a unique therapeutic challenge, since the usual rules of IOP reduction are often blurred. Even though this subgroup is often considered to be a continuum of primary open angle glaucoma (POAG), IOP is known to play a predominant role in POAG progression, potentially more vascular and other risk factors come into play, interacting with IOP across its entire range, in the pathogenesis of normal tension glaucoma. Shum et al outline the considerations for surgical decisions for normal or low tension glaucoma. They review current concepts of risk stratification, defining goal in terms of target pressure and IOP modulation as well as the strengths and weaknesses of available options, making the surgical decision-making easier for the reader.

Ortiz-Arismendi et al describe the results of their novel technique of implanting nonrestrictive glaucoma drainage devices. This technique of titrated ligature of Baerveldt tubes was found to be effective for controlling IOP during both the early and late postoperative phases in eyes with severe glaucomas.

In a digression from routine reviews on diagnosis and management of glaucoma, Bhartiya et al draw attention to the importance of mentorship programs for the acquisition of clinical and research skills as well as career growth for the individual glaucoma surgeon. The review aims to help the clinician to decide on how to choose a program that is best suited to his or her aspirations and abilities, can help to target and mitigate weaknesses, and help to formulate a rational and judicious career plan.

As always, we look forward to hearing from you.

Best wishes

**Shibal Bhartiya**

**Tarek Shaarawy**

**Tanuj Dada**

